# Associations Between Locomotion Scores and Specific Claw Lesions in Dairy Cows from Digital Dermatitis-Infected Herds

**DOI:** 10.3390/ani15192793

**Published:** 2025-09-25

**Authors:** Jasmin Laschinger, Anna-Lena Furtner, Birgit Fuerst-Waltl, Robert Pesenhofer, Johann Kofler

**Affiliations:** 1 Clinical Department for Farm Animals and Food System Science, Clinical Center for Ruminant and Camelid Medicine, University of Veterinary Medicine Vienna, 1210 Vienna, Austria; furtner97@gmx.at; 2 Department Agricultural Sciences, BOKU University, 1180 Vienna, Austria; birgit.fuerst-waltl@boku.ac.at; 3 Claw Trimming Practice Robert Pesenhofer, 8151 Hitzendorf, Austria; klaue234@gmail.com

**Keywords:** lameness, mobility score, claw disorders, digital dermatitis-associated claw horn lesions, interdigital hyperplasia, cattle

## Abstract

Lameness is a significant global welfare and economic problem in dairy farming. The objective of this study was to explore the relationship between various claw lesions and locomotion scores (LCS). A total of 491 cows from ten Austrian dairy farms were included in the study. Each cow’s gait was individually evaluated and an ascending locomotion score ranging from 1 to 5 was assigned. Subsequently, functional hoof trimming was conducted on all cows. During this procedure the claws were examined and all lesions electronically documented. For statistical analysis, the prevalence of each LCS was calculated and the distribution of LCS in relation to parity and lactation stage examined. Additionally, a simple chi-square test and a generalised linear mixed model (GLMM) were applied to identify associations between specific types of lesions and LCSs. The results from the GLMM showed significant associations (*p* < 0.05) between ‘other causes’, thin soles and interdigital hyperplasia with acute digital dermatitis on its surface (IH-DD-M2) and an LCS ≥ 2, between DD-associated claw horn lesions, thin soles, interdigital hyperplasia IH-DD-M2 and an LCS ≥ 3, and between DD-associated claw horn lesions, IH-DD-M2, and ‘infectious claw disorders’ and an LCS ≥ 4. The results emphasise the importance of early and accurate detection of lameness to promptly treat underlying painful claw lesions, thereby promoting improved animal welfare.

## Simple Summary

Claw lesions account for about 82% of cattle lameness cases, with the remaining cases attributed to injuries in the proximal limb. Although not all claw lesions result in lameness, there are a few disorders that are usually accompanied by lameness. This study of 491 cows from ten dairy farms aimed to identify which specific claw lesions are most associated with lameness. All cows were locomotion scored using an ascending five-point scale with locomotion scores (LCS) of 1–5. Functional hoof trimming was then performed, and all identified claw lesions were electronically recorded. The prevalence of each LCS was calculated and the distribution of LCSs in relation to parity and lactation stage assessed. A simple chi-square test and a generalised linear mixed model (GLMM) were applied to search for relationships between specific claw lesions and locomotion scores. Results from the GLMM revealed significant associations (*p* < 0.05) between digital dermatitis-associated claw horn lesions, interdigital hyperplasia with acute digital dermatitis, ‘infectious claw disorders’, thin soles and an LCS ≥ 2 and LCS ≥ 3, respectively. By better understanding these associations between a few painful claw lesions and LCS ≥ 2, targeted management measures can be implemented to detect lameness early, treat it promptly and correctly and implement preventive measures to promote improved animal welfare.

## 1. Introduction

Lameness in dairy cows is a global problem that significantly impacts animal welfare and contributes to economic losses [1,2,3]. Numerous studies have shown a substantial negative impact of lameness on milk yield, particularly in dairy cows with locomotion scores (LCS) ≥ 3 [2,4,5]. Additionally, a negative impact of lameness on fertility traits has been widely documented [6,7,8]. Milk losses and impaired fertility traits can vary, according to the type and severity of the claw lesions [2,5,7,8] and the persistence of pain-related lameness [1,9].

Premature culling of cows due to lameness represents the third highest cause of economic losses for Austrian dairy farms after mastitis and reproductive disorders [10]. On average, one case of lameness can result in economic losses of up to EUR 392 per cow per year [2]. Since the chances of recovery vary, depending on the claw disorder, correct and early diagnosis is particularly important [4,11,12].

Around 82% of all lameness cases in cattle are due to claw lesions and disorders of the digits [13]. The majority of lameness cases occur in the hindlimbs, with the outer claw being the most commonly affected [13,14,15]. Although claw lesions are the most common cause of lameness, the type and severity of the lesions strongly influence any noticeable change in gait [16,17,18].

Claw lesions that are usually accompanied by pain and lameness are referred to as ‘alarm’ lesions [19,20]. These include all ulcers (sole, toe and bulb ulcers), toe necrosis, white line abscess, interdigital phlegmon (foot rot) and infected, penetrating horn fissures, as well as moderate-to-severe swellings of the coronary band and bulb. The latter are highly indicative of the presence of deep digital sepsis [21]. In addition, acute stages (M2) of digital dermatitis (DD) in every location on the claw skin and all DD-associated claw horn lesions are also considered ‘alarm’ lesions [19]. In addition to the acute M2 lesions of DD, other stages include M1 (early stage), M3 (transition stage), M4 (chronic stage) and M4.1 lesion (chronic stage with a new M1 lesion). Detailed descriptions and illustrations of the various M-stages of DD can be found in the literature [22].

The locomotion scoring system, as described by Sprecher et al. [6], is a valuable tool for identifying lameness in dairy cows [19,23,24]. Studies report that the presence of a painful claw lesion in dairy cows is often linked to a higher locomotion score [17,18,23].

The main objective of this study was to determine if there is a relationship between different types of claw lesions and specific locomotion scores (LCS 1–5) in dairy cows. The study aimed to identify which specific claw lesions and claw lesion types are most frequently associated with lameness (LCS ≥ 2 and LCS ≥ 3).

## 2. Materials and Methods

### 2.1. Farms and Animal Data

For this clinical observational study, ten dairy farms located in the federal provinces of Lower Austria and Styria were selected as a convenience sample from an available pool of farms. The prerequisites were: a farm size of at least 25 cows, an average annual milk yield of at least 8500 kg, the farm location within a radius of ≤150 km from the University of Veterinary Medicine Vienna, and the farmers’ interest in participating in this study. An endemic DD infection was present on all ten farms. Farms 1 and 2 are regularly visited by clinic staff as part of veterinary student training. They practice preventive and therapeutic measures such as lameness checks at two-week intervals and immediate, proper treatment of cows with acute DD lesions at any location. In contrast, the other eight farms did not apply such measures.

Characteristics of the ten participating dairy farms are listed in Table 1. Data from locomotion scoring and hoof trimming documentation of 491 cows from these ten dairy farms were available for this analysis. Female cattle before their first parturition were not included in the evaluation. For each cow, the farm of origin, the ear tag number for precise identification, LCS, date of birth, age, breed, date of the last hoof trim, parity, stage of lactation, whether a block was already present on a claw, whether lesions were present on the limbs proximal to the claws and the respective lesions on all claws were recorded.

### 2.2. Locomotion Scoring

Between February 2023 and May 2023, a veterinarian (J.L.) with expertise in gait assessment and bovine orthopaedics conducted locomotion scoring of all cows on the ten dairy farms with free-stall housing at a single time point. The cows were initially restrained in the feeding fence and their back line was evaluated while standing. Subsequently, each cow’s gait and back line were individually assessed while walking in the alley. An LCS of 1–5 was assigned and recorded following the system of Sprecher et al. [6]. During locomotion scoring, clinical findings in proximal limb regions were also recorded.

### 2.3. Trimming and Documentation of Claw Lesions

Electronic records of claw lesions were created using the ‘Klauenmanager’ programme (SEG Informationstechnik GmbH, Bad Ischl, Austria) by two trained hoof trimmers during routine trimming visits of all cows examined in the ten herds. These experienced hoof trimmers were hired specifically for this study, as hoof trimming on these farms was typically performed by the farmers themselves or other personnel who did not record claw lesions. Both hoof trimmers had participated in an inter-observer reliability test for the correct detection of claw lesions necessary for a previous study and achieved a weighted Cohen’s kappa value of >0.82 [19].

Hoof trimming and recording of claw lesions were performed on the same day (*n* = 3), the following day (*n* = 5), three days (*n* = 1) or seven days (*n* = 1) after gait assessment. In these protocols, all claw lesions were documented using the terminology from the ICAR Claw Health Atlas [25] and its two appendices [22,26]. Within the electronic documentation system, each claw lesion was recorded separately for all eight claws based on the ten-zone system [27]. Three severity scores were used for many of the claw lesions [27].

Since these farms were exclusively dairy farms with endemic DD, we assumed, based on the same characteristic surface morphology of the exposed corium and the same pungent smell as is common in M2 lesions on digital skin [26,28], and the results of a recent study [29], that all claw horn lesions with exposed corium were associated with DD. However, we did not take samples for quantitative and qualitative PCR to detect DD-specific *Treponema* spp. This category included all types of ulcers (sole ulcer, toe ulcer, bulb ulcer), toe necrosis, white line lesions with severity scores 2 and 3 (also known as white line abscesses) and perforating horn fissures.

### 2.4. Statistical Analyses

The collected data were transferred to Microsoft Excel 2020 (Microsoft Corp., Redmond, WA, USA) for statistical analysis. The prevalence for each LCS (1–5) was calculated by dividing the number of cows per LCS by the total number of animals examined. This calculation was also performed for animals with an LCS ≥ 2 and an LCS ≥ 3.

Additionally, the distribution of LCS was analysed in relation to the cows’ parity, and stage of lactation, such as early lactation (≤100 days in milk; DIM), mid lactation (101–200 DIM) and late lactation (>200 DIM). Mean, standard deviation (SD), median, minimum (Min) and maximum (Max) values of claw lesions were determined.

We considered three severity scores for the claw lesions with some exceptions as outlined in the documentation programme [27]. We evaluated the number of recorded claw lesions per animal, counting all instances, even if multiple occurrences were found in the same cow.

The prevalence of lesions was evaluated separately for the forelimbs and hindlimbs, as well as for lateral and medial claws. Additionally, the prevalence of so-called ‘alarm’ lesions and their distribution on the limbs were assessed. This included all acute DD stages at any location, including M2 stages on the top of an interdigital hyperplasia (IH-DD-M2), all DD-associated claw horn lesions, inflammatory swelling of the coronary band and bulb and interdigital phlegmon (foot rot). Furthermore, the prevalence of ‘alarm’ lesions was assessed with LCS 1 (non-lame).

Based on these data, an intra-herd prevalence was calculated to indicate how frequently at least one claw lesion occurs within a single herd. The intra-herd prevalence was calculated using the following formula:

$$Intra-herdprevalence=\frac{Number\;of\;cows\;with\;at\;least\;one\;claw\;lesion\;in\;the\;herd}{Total\;number\;ofcows\;in\;the\;herd}\times 100$$

The cow prevalence describes the frequency of at least one claw lesion considering only the highest documented severity score of the respective lesion among all examined cows. Cow prevalence was calculated using the following formula:

$$Cow\;\;\;prevalence=\frac{\begin{array}{c} Number\;of\;cows\;showing\;at\;least\;one\;claw\;lesion\;\\ considering\;the\;highest\;severity\;score\;only\; \end{array}}{Total\;number\;of\;cows}\times 100$$

To investigate the statistical relationship between various claw lesions and types of claw lesions and LCS ≥ 2, LCS ≥ 3 and LCS ≥ 4, a simple chi-square test was performed using the procedure freq in SAS 9.4 (SAS Institute Inc., Cary, NC, USA). The findings were reported as odds ratios (ORs) with corresponding 95% confidence intervals to quantify the strength and direction of the associations. In cases where the assumptions of the chi-square test, such as minimum expected cell counts, were not met, Fisher’s exact tests were additionally employed to ensure the validity of the statistical analysis.

Potential influencing factors such as parity and lactation stage were also included in the analysis. Therefore, a generalised linear mixed model (GLMM) was constructed using proc glimmix in SAS 9.4 (SAS Institute Inc., Cary, NC, USA). The model included all claw lesions, parity (divided into five classes: first to fifth and higher lactations), and stage of lactation (divided into three classes: early, mid and late lactation) as fixed effects, and the farm as a random effect.

As LCS is recorded on the cow level rather than the individual claw level, all calculations regarding the relationship between claw lesions and LCS are based on data with the highest severity score per lesion observed per cow. For example, if a cow had two heel horn erosions (HHE) with a score of 3 and six HHE with a score of 2 or 1, only one of the HHE lesions with the severity score of 3 was used for the statistical calculation. This approach ensured consistency between the levels of data aggregation and simplified the analysis while retaining the most relevant information about claw lesions.

A significance level of α = 0.05 was chosen for all analyses. A cross-tabulation of the results was created for visualisation. In this analysis, the various claw lesions are categorised using established abbreviations (Table 2).

## 3. Results

### 3.1. Mean Lameness Prevalence and Prevalence of Individual Locomotion Scores

The mean lameness prevalence across all ten dairy farms was 59.3% (LCS ≥ 2) (SD: 24.1%, median: 60.6%, min: 32.5%, max: 97.7%) and 17.7% (SD: 22.6%, median: 9.0%, min: 0.0%, max: 72.1%) when clinical lameness was defined as LCS ≥ 3.

The breakdown of the individual locomotion scores revealed that 200 cows showed no signs of lameness (LCS 1: 40.7%), 204 cows were mildly lame (LCS 2: 41.5%), 64 cows had LCS 3 (13.0%) and 23 cows (4.7%) had LCS 4. No cows were diagnosed with LCS 5; therefore, the cows were categorised into four groups (LCS 1, LCS 2, LCS 3, LCS 4). In total, 70.1% (*n* = 204) of all lame cows exhibited an LCS 2. Notably, farms 1 and 2 did not have any animals with an LCS ≥ 3.

### 3.2. Distribution of Cows by Age and Locomotion Scores

The cows ranged in age from 2.0 to 12.0 years, with a mean age of 4.6 years (SD: 2.0; median: 4.0). The largest age group consisted of three-year-old cows (*n* = 123), followed by four-year-old cows (*n* = 101) and five-year-old cows (*n* = 93). The remaining cows were older than five years.

### 3.3. Distribution of Cows by Parity and Locomotion Scores

**Figure 1:**
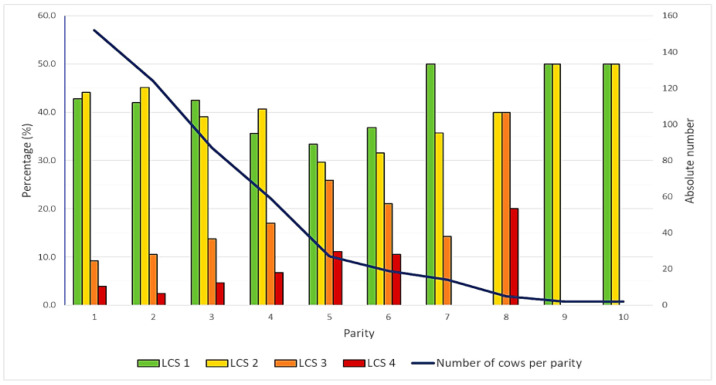
Bar chart showing the percentage of cows in each parity categorised by their locomotion score (LCS 1–4). The black line represents the total number of cows per lactation number.

Mean parity was 2.7 (SD: 1.8; median: 2.0; min. 1.0; max. 10.0). Most of the cows were in their first (*n* = 152; 31.0%) or second lactation (*n* = 124; 25.3%). The number of cows decreased with increasing parity. A shift in the distribution of the individual LCSs was also observed with increasing parity. In the first three lactations cows mainly had an LCS 1 (41.9%–42.8%) or 2 (39.1%–45.2%). However, the proportion of animals with an LCS of 3 and 4 increased steadily until the fifth and sixth lactations (Figure 1).

### 3.4. Distribution of Cows by Stage of Lactation and Locomotion Scores

The distribution of cows by stage of lactation showed that 31.6% were in the first 100 DIM, 27.7% in mid lactation (101–200 DIM) and 40.7% in late lactation (>200 DIM). Allocation, according to LCSs, showed that in all stages of lactation most cows had an LCS 1 (first 100 DIM: 45.2%; 101–200 DIM: 34.6%; >200 DIM: 41.5%) or 2 (first 100 DIM: 42.6%; 101–200 DIM: 43.4%; >200 DIM: 39.5%). As the lactation stage increased, the number of cows with LCS ≥ 3 also increased. While in early lactation only 19 out of 155 cows (12.2%) had an LCS ≥ 3, in mid lactation 30 out of 136 (22.1%) and in late lactation 38 out of 200 cows (19.0%) had an LCS ≥ 3.

### 3.5. Prevalence of Claw Lesions at Cow Level

The calculated prevalences of claw lesions consider the highest documented severity score for each lesion. The highest prevalences were observed for claw horn lesions without an exposed corium (58.0%), heel horn erosion (54.4%), claw deformations mainly composed of concave dorsal walls (23.2%), acute digital dermatitis (15.3%) and DD-associated claw horn lesions (10.4%). Other M-stages were diagnosed in a total of 38 cows (7.7%; with M1 stages in 16 cows and M4 stages in 22 cows), and these cows had no M2 lesions. Interdigital hyperplasia with acute DD on top was observed in 27 cows (5.5%). All other lesions showed prevalences between 10.8% and 1.8% (Table 3).

In the majority of cows (*n* = 80; 16.3%), eight claw lesions per animal were documented during the one-time claw health data recording, followed by four lesions (*n* = 58; 11.8%) and ten lesions (*n* = 52; 10.6%). No claw lesions were documented in 5.7% of cows, while 20 or more claw lesions were found in 1.4% of cows. The distribution of the lesions showed that just over half (56.2%) of the cows had between four and ten claw lesions. In total, 2010 claw lesions (45.0%) were documented on the forelimbs and 2452 (55.0%) on the hindlimbs. Most of the claw lesions on the hindlimbs occurred on lateral claws (*n* = 1544; 63.0%). In contrast, the distribution of claw lesions on the lateral and medial claws of the forelimbs was almost equal (*n* = 1002 vs. 1008) (Table 4).

A variety of claw lesions were documented in cows with LCS 1 (*n* = 200; 40.7%): HHE in 97 cows (48.5%), claw horn lesions without exposed corium in 103 cows (51.5%), claw deformations in 38 cows (19.0%), interdigital hyperplasia in 10 cows (5.0%), thin soles in 3 cows (1.5%), early and chronic stages (M1, M4) of DD in 18 cows (9.0%), acute DD in 25 cows (12.5%), and DD-associated claw horn lesions in 14 cows (7.0%) (Table 5).

A total of 215 ‘alarm’ lesions were diagnosed in 185 (37.7%) out of 491 cows. These cows also exhibited interdigital hyperplasia with acute DD on top and thin soles, which, are now considered ‘alarm’ lesions, based on these results.

In total, ‘alarm’ lesions were recorded in 43 cows (21.5%) scored with LCS 1, regardless of the location of the lesions. The distribution of ‘alarm’ lesions across the locomotion scores LCS 1, LCS2, LCS 3 and LCS 4 is shown in Table 6.

In 25 cows (5.1%), one or more of these ‘alarm’ lesions were located on the lateral claws of both hindlimbs. Among these 25 cows, seven had LCS 2, nine had LCS 3 and nine had LCS 4.

In 19 (3.9%) out of 23 cows, the following diagnoses were made in proximal limb regions, representing ‘other causes’: abscesses, haematomas, muscle trauma with severe swelling, tarsal bursitis, calcaneal bursitis, healed fracture of the coxal tuber and severe udder swelling in cases of acute mastitis. Four cows from this group ‘other causes’ had a block applied to medial hind claws for treatment of white line abscesses.

### 3.6. Intra-Herd Prevalence and Cow Prevalence of at Least One Claw Lesion

Analysis of the intra-herd prevalence revealed that a total of 97.1% (ranging from 78.4% to 100%) of cows had at least one claw lesion. Compared to the other nine farms, farm 3 had a lower prevalence of claw lesions, with 78.4% of cows affected by at least one claw lesion and 25 cows showing no lesions.

A total of 28 cows (5.7%) were observed without claw lesions, while 463 cows had at least one claw lesion. The overall cow prevalence of at least one claw lesion was 94.3%.

### 3.7. Association Between Specific Claw Lesions and Locomotion Scores

The simple chi-square test showed a statistically significant association (*p* < 0.05) between O-caus, CHL-wexcor, DD-ass, IH-DD-M2, IH, HHE and TS, and an LCS ≥ 2. There was also a statistically significant association between O-caus, C-deform, CHL-wexcor, Infect, DD-ass, IH-DD-M2, IH, HHE and TS and an LCS ≥ 3 (Table 7).

Additionally, there was a statistically significant association between O-caus, DD-ass, CHL-wexcor, Infect, DD-ass, IH-DD-M2, IH, HHE and an LCS ≥ 4. The OR of having an LCS ≥ 2, LCS ≥ 3, or LCS ≥ 4 was 1.9 times, 4.4 times or 5.3 times higher in cows with DD-associated claw horn lesions, respectively, than in cows without these lesions.

The OR of having an LCS ≥ 2, LCS ≥ 3, or LCS ≥ 4 was 5.6 times, 6.1 times, or even 19.5 times higher in cows with infectious claw disease (without DD) than in cows without this lesion. The OR of having an LCS ≥ 2, LCS ≥ 3, or LCS ≥ 4 was approximately 19.5 times, 6.8 times, and 12.6 times higher, respectively, in a cow with IH-DD-M2 than in cows without this claw lesion (Table 7).

When calculating the generalised linear mixed model, a statistically significant association (*p* < 0.05) was found between the disorders O-caus, IH-DD-M2 and TS and an LCS ≥ 2 between the lesions DD-ass, IH-DD-M2, IH, and TS and an LCS ≥ 3 and between infectious claw disorders, DD-ass, and IH-DD-M2 and an LCS ≥ 4 (Table 7).

The OR for having an LCS ≥ 2, LCS ≥ 3 or LCS ≥ 4 was 1.5 times, 4.0 times and 5.3 times higher, respectively, in a cow with DD-associated claw horn lesions than in cows without this claw lesion.

The OR for an LCS ≥ 2, LCS ≥ 3 or LCS ≥ 4 being present, was approximately 17.6 times, 3.4 times, and 9.1 times higher, respectively, in a cow with interdigital hyperplasia and acute digital dermatitis on top (IH-DD-M2) than in cows without this lesion using this calculation method (Table 7).

There was no statistical significance for an LCS ≥ 2 in cows with an acute DD, neither in the calculation using the chi-square test nor using GLMM (Table 7). No significant associations with specific LCSs were observed in any of the three models (LCS ≥ 2, LCS ≥ 3, LCS ≥ 4) for either lactation stage (*p* = 0.4355, 0.3919 and 0.6257) and parity (*p* = 0.9056, 0.0504, and 0.5573).

For LCS ≥ 3, the *p*-value for parity was close to the significance limit (*p* = 0.0504). Cows in their fifth lactation and later tended to have a higher risk of lameness than cows in the first three lactations (OR: 3.769 for 5th and higher versus 1st lactation).

By directly comparing the incidence of different claw lesions in the same cows using crosstabulation, it was shown that, e.g., HHE (2.6% to 66.7%) and claw horn lesions without exposed corium (2.5% to 62.5%) frequently occurred in addition to other lesions. It should also be noted that ‘other causes’ of lameness (O-caus) occurred in combination with different claw lesions in 8.7% to 69.6% of cases (Table 8).

## 4. Discussion

In our study, the relationship between locomotion scores 1–4, according to Sprecher et al. [6], and specific claw lesions in Austrian dairy cows from herds infected with DD was investigated for the first time. We hypothesised that certain painful claw lesions would be linked to a certain level of lameness, as described by other authors [15,17,18]. While our sample size, with 491 dairy cows from ten Austrian dairy farms, was smaller compared to other studies with similar objectives [18,24,30], it was sufficient for a robust statistical analysis. The results of the two analysis methods—simple chi-square/Fisher’s exact tests and generalised linear mixed models—were consistent and showed no contradictory findings.

Since it is recognised that the observer’s experience and the environment might impact visual gait assessment [23,24], locomotion scoring was conducted by a veterinarian with extensive experience in bovine orthopaedics and gait assessment, ensuring consistent evaluation across the entire sample [31]. Likewise, the claw lesions were assessed by two well-trained hoof trimmers, who had achieved very good kappa values of > 0.82, indicating almost perfect agreement [32] in an inter-observer reliability test in a previous study [19]. Therefore, the claw health data documented by these hoof trimmers could be considered of high quality [32,33]. Furthermore, the claw lesions of the cows on eight farms were recorded either on the same day or the day after locomotion scoring. Only on the remaining two farms did hoof trimming and documentation take place three or seven days later, respectively. Due to this relatively good temporal coincidence, the connection between the recorded LCS and claw lesions was largely maintained. Certainly, for the objective of this study, it would have been better to perform hoof trimming and document claw lesions on all farms on the same day or at least on the following day. However, this was not possible on two farms due to organisational reasons. It should be considered that infectious claw diseases such as acute DD and interdigital phlegmon can develop within a few days only. The development of claw horn lesions is known to cause subclinical lameness, which is frequently defined as LCS ≤ 2 [2,5], and a reduction in milk yield up to eight weeks before their visual detection during claw examination, without any noticeable lesions being diagnosed on the claw at previous times [2,5]. In similarly designed studies, hoof trimming and documentation of claw lesions were also performed on the same day [16,24], one day later [18], or ‘shortly after’, without specifying this information [30].

Our assumption that all claw horn lesions with exposed corium in cows from dairy farms with an endemic DD status are infected with digital dermatitis specific *Treponema* spp. was strongly supported by the clinical findings, the consistent pungent smell, and the characteristic surface morphology of the exposed corium, as seen in acute M2 lesions on the digital skin [26,28]. This was further validated by the results of a recently published cross-sectional study [29]. Dias et al. [29] demonstrated that DD-associated *Treponema* spp. were exclusively detected in DD-affected lesions, DD-foot and other skin lesions, as well as in healthy skin of cows in DD-affected herds. In contrast, non-DD-specific *Treponema* spp. were found in samples from cows in both DD-negative and affected herds [29]. Based on this evidence, we assumed involvement of DD-specific *Treponema* spp. in lesions with exposed corium in herd with endemic DD-infection, primarily to ensure consistency in lesion classification. However, we acknowledge that our study did not assess whether the presence of DD-associated *Treponema* spp. influences the severity of the lesion, pain, or locomotion. Importantly, the main findings of our study, particularly the high prevalence of claw horn lesions with exposed corium in mildly lame cows, remain valid regardless of Treponema status. Although claw horn lesions with an exposed corium always represent a possible cause of lameness, whether the infection is with DD-specific treponemes or with other bacteria [12,14,17,26,28].

Based on the results of several studies, it can be inferred that only a few specific claw lesions, such as sole ulcers, white-line lesions, double soles, sole haemorrhages, interdigital hyperplasia, acute digital dermatitis, and interdigital phlegmon (foot rot), result in noticeable changes in gait after reaching a certain level of severity [9,14,15,18]. This suggests that the presence or absence of visually detected lameness has limited significance for identifying a specific claw lesion [15,18,30].

Our results validate that only a few specific claw lesions usually associated with pain, were significantly correlated (*p* < 0.05) with an increased LCS and therefore clinical lameness. Specifically, claw horn lesions such as all types of ulcers, white line abscesses, perforating horn fissures (whether DD-associated or not), interdigital hyperplasia with and without acute DD on top (IH-DD-M2), and thin soles showed higher odds ratios for an LCS ≥ 2 (with an OR between 1.9 and 19.5) and LCS ≥ 3 (with an OR between 3.7 and 6.8). This was also true for infectious claw disorders and other (proximally located) lameness causes. The odds ratios for lameness occurrence in cows with LCS ≥ 2 varied depending on the method of calculation. The simple chi-square test resulted in higher ORs compared to the GLMM.

The results of our trial are consistent with those of other studies in which painful claw lesions, such as sole ulcers [14,15,16,17,18,24,34] and white line abscesses [18,20,23,24], correlated with significant alterations in gait. It was reported that cows with sole ulcers were significantly more likely to have an asymmetrical gait pattern, reduced weight bearing and an arched back line [14,16]. In our study, all sole ulcers, toe ulcers and white line abscesses were classified as DD-associated claw horn lesions. This classification was made because all the cows involved were housed in herds with endemic DD infection. Jewell et al. [30] reported that cows with sole ulcers and acute DD (M2 stage) more often showed uneven weight distribution or unloading of a limb. Data from our study complement the previous literature by showing that, in addition to the above mentioned usually painful claw lesions, known as ‘alarm’ lesions [19,20], other lesions, such as interdigital hyperplasia with acute DD on their surface and thin soles were also associated with an LCS > 1, LCS > 2 and LCS > 3, indicating mild-to -moderate lameness. The combined occurrence of interdigital hyperplasia with acute DD on its surface in the interdigital space (IH-DD-M2) showed the highest determined OR of 19.5 for LCS > 1 and 12.6 for LCS > 3.

Statistical calculations using the GLMM mostly confirmed these results. Significant associations were found between DD-associated claw horn lesions (OR: 4.0; 95% CI 1.6–9.9), interdigital hyperplasia (OR: 3.0; 95% CI 1.2–7.5), the combination of interdigital hyperplasia with acute DD (OR: 3.4; 95% CI 1.1–10.9), thin soles (OR: 7.2; 95% CI 1.1–47.8), with an LCS ≥ 3 and between DD-associated claw horn lesions (OR: 5.3; 95% CI 1.3–21.2), the combination of interdigital hyperplasia with acute DD (OR: 9.1; 95% CI 2.3–36.4), and infectious claw disorders (OR: 10.0; 95% CI 1.7–57.7) with an LCS ≥ 4. This confirms that on farms with endemic DD, DD-associated claw horn lesions and the combination of IH with acute DD are particularly important claw disorders for causing moderate and severe lameness (LCS ≥ 3). This has also been reported in previous studies [18,23,27].

Our study revealed that even in cows without lameness (40.5%), various claw lesions were also present. They were not typically considered severe, such as HHE, claw horn lesions without an exposed corium, claw deformations, interdigital hyperplasia, M1 and M4 stages of DD, and thin soles. However, surprisingly 21.5% of the cows with an LCS 1 had ‘alarm’ lesions, including acute DD in 25 cows, DD-associated claw horn lesions in 14 cows, and thin soles in 3 cows (see Table 6). These lesions are usually associated with pain and lameness [19,20,28,35]. Potential reasons for the LCS 1 rating in these cases could be the low-pressure load at the site of the acute DD-M2 lesion during walking, the early stage of a DD-associated claw horn lesion, a lower body weight of cows with thin soles, and the individual pain tolerance of the cows [1,30]. However, another explanation could be inaccurate locomotion scoring, which is still somewhat subjective despite thorough examiner training [17,30].

Neither the chi-square test nor the GLMM, which accounted for farm-specific effects, indicated a statistically significant association between the presence of acute DD and an LCS ≥ 2, with ORs of 1.4 and 1, respectively. This unexpected result could be due to the location of the acute DD-M2 lesion, the presence of acute DD lesions on both hindlimbs simultaneously and the individual pain sensitivity of the cows [1,14,30]. Although acute M2 lesions are often painful to touch, they do not always result in clinical lameness [36]. These authors reported that 61% of cows with acute DD lesions were not lame, 26.5% were mildly lame (LCS 2), 32.6% were moderately lame (LCS 3) and only 10.2% were severely lame (LCS ≥ 4) [36]. Other studies have also reported no significant association between the occurrence of DD and LCS ≥ 2 [14,16,18].

It is striking that the prevalence of lameness and acute DD, as well as DD-associated claw horn lesions, was high in the ten farms studied, even though most farms performed hoof trimming two-to-three times annually. One possible explanation for this finding is that functional hoof trimming every four-to-six month alone is not an efficient measure to reduce significantly the prevalence of lameness caused by infectious claw disorders such as acute DD and interdigital hyperplasia with acute DD [37,38]. To reduce the prevalence of DD, improvements in the quality of cubicle bedding, cleanliness and dimensions of the walking areas, a reduction in stress factors, the implementation of lameness checks at two-week intervals and the immediate and proper treatment of cows with acute DD lesions at any location, as well as regular measures to prevent M1 and M4 lesions from developing into acute M2 lesions are required [39,40]. However, such measures had been practised on farms 1 and 2, where no cows scored an LCS ≥ 3. Moreover, a significant reduction in claw horn lesions has been reported when functional hoof trimming is performed at short intervals of approximately 4.5 months and at dry off [37,38,41].

Our results suggest that not every claw lesion leads to recognisable lameness. As shown in Table 5, 19.0% of claw horn deformations, 51.5% of claw horn lesions without an exposed corium (including sole haemorrhages, double soles, and white-line lesions of severity score 1 indicating white line separation), 48.5% of HHE, and 5.0% of interdigital hyperplasia were recorded in cows scored with LCS 1 (non-lame). Specifically, when these lesions are located superficially in the claw horn, such as HHE, often in sole haemorrhages and double soles, and when they are located at anatomical sites of the claw that are subject to little or no pressure (small lesions on the interdigital skin and skin proximal to the claw horn), lameness (LCS ≥ 2) rarely occurs when the animal is standing or walking [18,42]. In our study, HHE was the second most frequently documented claw lesion, which was diagnosed in 54.4% of cows. A statistically significant relationship (*p* = 0.0301) was identified between the presence of HHE and an LCS ≥ 2 only when using the chi-square test. However, this association was not observed with GLMM. This suggests that HHE, characterised by macerated superficial horny layers and often linked with laminitis, is generally not associated with pain and therefore rarely leads to lameness on its own [14,42]. Nevertheless, this finding should not be ignored in the context of claw health monitoring, as the presence of HHE of severity scores 2 and 3 can increase the risk of subsequent, particularly infectious, claw skin disorders [14,20,39]. Several authors have concluded from their studies that there is no increased probability (OR) for lameness in cows with HHE [14], and that the presence of severe HHE was not associated with an increased lameness risk (*p* = 0.08) [43]. In our study, the GLMM showed that HHE had no statistically significant increased probability of being associated with non-lame (LCS 1) or lame cows (LCS ≥ 2) with an OR of 0.9 (95% CI 0.5–1.6). This suggests that HHE is unlikely to be a cause of lameness.

Lameness in dairy cows can have causes other than claw lesions. In a recently published study, approximately 18% of all cases of lameness in beef and dairy cattle were found to be localised in proximal limb regions [13]. In our study of dairy cows, we found that ‘other causes’ accounted for 3.9% of cases excluding cows where a block had been glued. They included a variety of disorders such as abscesses, haematomas, muscle trauma with severe swelling, tarsal bursitis, calcaneal bursitis, healed fracture of the coxal tuber, and severe udder swelling in cases of acute mastitis as also reported elsewhere [44]. This result emphasises the importance of conducting an orthopaedic examination of the proximal regions of the lame limb and, in some cases, a general clinical examination in cattle with lameness in addition to a careful examination of the claws. This is especially crucial if the degree of lameness [1,3,13] and the type of lameness (supporting limb or swinging limb lameness) cannot be fully explained by the detected claw lesions alone [45].

The presence of claw lesions on both hind- or forelimbs of a cow, especially painful lesions like ulcers, white line abscesses and interdigital hyperplasia associated with acute DD and interdigital phlegmon, often results in a situation where clear lameness associated with a higher LCS of one limb is less easily identifiable. Instead, cows with painful claw lesions on both hind- or forelimbs tend to exhibit a ‘stiff’ gait [1,46], making lameness identification more challenging through visual gait assessment and kinematic measurement methods [34]. In the current study, 16.3% of the cows had eight lesions spread across multiple claws. Furthermore, 25 cows exhibited ‘alarm’ lesions on the lateral claws of both hindlimbs showing a ‘stiff’ gait with still recognisable LCSs ranging from 2 to 4 according to the method of Sprecher et al. [6]. This method of locomotion scoring may not be well-suited to accurately describe bilateral lameness. This highlights that cows with painful claw lesions on two opposite limbs may not always receive a higher LCS, possibly due to their limited ability to compensate for their lameness, resulting in lower perceived severity [17,34]. Numerous other researchers have reported that a high percentage of cows, ranging from 26% to 82.2%, exhibited more than one claw lesion, with differences observed between regions, housing types, herds and breeds [30,47,48,49].

The stage of lactation (trimesters 1, 2, and 3) did not show a statistically significant correlation (*p* > 0.3919) with LCS in any of the three models, as has also been found by others [24]. However, studies with a significantly larger number of cows did find significant correlations between body condition score and digital fat cushion thickness. Both these parameters show lower values in the negative energy balance phase in early lactation, specifically during the first trimester, and LCS ≥ 3 [9,50].

A similar lack of correlation was also observed between parity and LCS. However, in the LCS ≥ 3 model, a trend was evident (*p* = 0.0504) that cows with parity ≥ 5 showed higher LCSs compared to cows with lower parity. Other authors who evaluated data from a larger number of cows reported significant associations (*p* < 0.001) between the severity and prevalence of lameness or claw horn lesions and higher parity, with ORs of > 2.53 (95% CI 1.57–4.08) [24,37,49,50]. This is explained by various factors, such as the detrimental impact of the flooring system, inadequate dimensions and quality of the lying areas, overcrowding, prolonged standing times, poor hygiene, inadequate lameness management, among others [11,12,37,43,47,51,52]. Changes in the composition and thinning of the shock-absorbing digital fat cushions, as well as the development of bone proliferations on the flexor tubercle of the pedal bone, increase with parity [9,53,54].

The results of our study are largely consistent with findings from previous publications that have also described a correlation between lameness and specific claw lesions [14,18,23,24,30]. However, these studies have revealed differences in the relative distribution of claw lesions, which can be attributed to various factors such as different housing systems, management and feeding practices and environmental conditions on the farms studied [18,30,52].

In the studies mentioned, researchers established a threshold of LCS ≥ 3, above which clinical lameness is agreed [9,20,24]. For instance, a study from Germany found lameness prevalences ranging from 23.1% to 39.1%, depending on the region, with only animals scoring 3 or higher on the LCS scale being classified as lame [55]. Using this definition of clinical lameness (LCS ≥ 3), the mean lameness prevalence in all ten herds was 17.7%. However, when the threshold value of LCS ≥ 2 was chosen, the lameness prevalence skyrocketed to 59.3%. Sprecher et al. [6] noted in their influential study that an LCS of 2 indicates clinically recognisable lameness. Some researchers define clinical lameness as LCS ≥ 3 [24,42,56]. However, it is important to reconsider this threshold, because cows with an LCS of 2, indicating mild lameness, should be identified early for successful treatment. This is beneficial both economically and ethically [4,11,12]. While numerous studies stress the importance of preventing severe lameness (LCS ≥ 4) to enhance efficiency and animal welfare in herds facing lameness problems, achieving this goal requires early detection and proper treatment of cows with LCS 2 [4,11,19,38].

In addition to visual locomotion scoring [6,17,57], as used in our study, numerous automated techniques for lameness detection have been described in recent years [46,58,59]. However, until these methods can be easily and inexpensively implemented and operated by farmers in the barn, visual lameness detection remains indispensable [31,57].

For reliable detection of lameness on farms, it is crucial to implement a standardised lameness scoring system. This should be performed regularly at two-week intervals [4,57], ideally in cooperation with the farm veterinarian and the hoof trimmer [5,31,55]. Several authors [1,4,57] emphasise the importance of establishing regular gait assessments and claw inspections as key components for monitoring claw health. Again, ideally, these assessments should occur at two-week intervals [1,4,57], especially for all cows being dried off, and cows in early lactation between 60 and 100 DIM [19,31].

Early and accurate detection of lameness by farmers could be greatly enhanced through repeated training in lameness assessment. A recent Austrian study compared farmers’ assessments of lameness prevalence on their farms with expert recorded data [31]. The findings of this study, along with others indicated that lameness prevalence on many dairy farms is often underestimated by 20% to 30% [55,60].

The results of our study showed that 59.3% of the cows examined across the ten farms had an LCS ≥ 2. Looking at the farms individually, the lameness prevalence (LCS ≥ 2) was over 30% in all herds, with half of the farms even exceeding the lameness prevalence (LCS ≥ 2) by over 50%. These prevalences are significantly higher than the recommended threshold of 10% [61] or the lameness incidence of farms in the tenth percentile when benchmarking claw health [19]. Similarly high prevalences were found in this benchmarking study from Austria, which reported a mean annual lameness incidence of 39.4% (LCS ≥ 2) and 14.6% (LCS ≥ 3) on 99 dairy farms [19]. In this context, the legitimate question arises as to why such a high lameness prevalence was present on these farms if the lameness threshold is defined as LCS ≥ 2. Potential risk factors for high lameness prevalence on the farms studied are most likely due to environmental and feeding-related causes, but mainly inadequate or even non-existent professional claw health management in eight of these farms [43,51,52] and a lack of or inadequate lameness control [31,55,60]. However, these potentially relevant variables were not assessed in the present study.

Analysis of the claw lesions documented showed a clear clustering in the hindlimbs, with the lateral claws (left lateral claws: *n* = 777; right lateral claws: *n* = 767) being significantly more frequently affected than the hind medial claws or the claws of the forelimbs. These results are consistent with numerous studies concerned with the epidemiology of claw disorders [13,14,30,47,52]. This striking distribution of claw lesions on the hind lateral claws can be well explained by the measured higher biomechanical loads [37,62] and anatomically, namely that the bones of the lateral digit are 2 to 3 millimetres longer than those of the medial digit [63].

Some limitations need to be noted about the present study. The primary limitation seems to be the relatively small number (491) of cows examined. In comparison, other similarly designed studies have included far greater numbers of cows, e.g., 1056 cows [16], 1098 cows [18], 1180 cows [14], 1340 cows [23], 1950 cattle [52] and 2569 cows [24]. Jewell et al. [30] used a similarly sized population of 401 cows and 557 lameness observations, while Hässig et al. [15] included only 56 cows in a study with a similar research objective, albeit with lameness assessments at three different time points.

The sample size of 491 cows was determined by a previous study addressing a different research objective [31] and therefore could not be altered. The sample size and the low prevalence of some claw lesions, such as double soles, ulcers, white line abscess, interdigital hyperplasia, thin sole, horn fissures and interdigital phlegmon in the cows examined from the ten farms, as well as possibly the small number of farms included, could also impact the results of the selected statistical models. However, this argument must be countered by the fact that analyses of electronically documented claw lesions during hoof trimming of 17,838 cows from 512 Austrian herds in 2020 [19] and of 28,638 cattle from 526 dairy farms in Austria over a period of ten years [49] showed similarly low prevalences and incidences for these particular claw lesions.

A potential impact on the results of our study could be the high proportion (70.1%) of mildly lame cows (LCS 2) among the total number of lame cows, corresponding 41.5% of all 491 examined cows. This may make it statistically more challenging to attribute specific claw lesions to higher locomotion scores [15,24], because the percentage of cows with an LCS of 3 and higher was only 17.7% in our study. However, it is important to note that in many commercial dairy herds, as well as in numerous studies, the distribution of LCS ≥ 2 resembles a pyramid (referred to as the ‘lameness score pyramid’), with a large number of LCS 2, a smaller number of LCS 3, yet fewer LCS 4, and very few LCS 5 [18,19,23,24,42]. Such a pyramidal distribution of LCS 2, LCS 3 and a small number of LCS 4 at the top was also observed in the current study.

Another limitation of our study was that the cows on the ten farms were subjected to a gait assessment and a claw examination only once within a four-month period in the spring. This was comparable to the study by Tadich et al. [18], where data collection also extended to four months. However, in other studies, with similar research designs, gait observations and claw examinations were conducted up to three times consecutively, for instance, at drying off, during the first week after calving and two months after calving [15], or data collection spanned a total of six months [52] or even 12 months [30]. This allows for better consideration of seasonal fluctuations in the prevalence of lameness and the type of individual claw lesions and thus different risk factors. Furthermore, potential risk factors for lameness on the farms studied were not recorded.

## 5. Conclusions

Our study confirmed a close association between a few specific painful claw lesions such as ulcers, white line abscesses, interdigital hyperplasia with acute DD on the top, ‘infectious claw disorders’ and thin soles (collectively referred to as ‘alarm’ lesions) and higher locomotion scores in dairy cows. The results demonstrate that only about five or six claw lesions, specifically those mentioned above, are frequently associated with lameness. However, lameness can also occur without obvious claw lesions. In these cases, the examiner must use a hoof tester to determine if one of the claws is painful. If not, the proximal limb regions should be examined for potential causes of lameness. By better understanding these associations between a few painful claw lesions and LCS ≥ 2, targeted management measures can be implemented to detect lameness early, provide treatment, or take preventive measures.

## Figures and Tables

**Table 1:** Characteristics of the ten participating dairy farms.

Farm	Number of Cows Analysed (Cow Number in the Herd)	Mean Annual Milk Yield (kg)	Dominant Breed ^1^	Mean Age of Cows in the Herd (Years)	Hoof Trimming Frequency per Year ^2^
1	40 (73)	9729	FV	5.5	2–3 times (F)
2	37 (40)	9427	HF	4.3	3 times (HT)
3	106 (123)	13,801	HF	4.1	3 times (HT)
4	70 (83)	10,195	FV	6.3	2 times (F)
5	34 (39)	10,668	HF	5.1	Every 9 months (HT)
6	27 (27)	10,539	FV	6.5	2 times (HT)
7	44 (46)	10,732	FV	5.0	2 times (HT)
8	46 (52)	10,683	FV	5.1	2 times (HT)
9	44 (48)	10,058	FV	5.5	3 times (HT)
10	43 (47)	10,453	BS	5.5	3 times (HT)

^1^ FV: Fleckvieh (dual purpose Simmental); HF: Holstein-Friesian; BS: Brown Swiss. ^2^ F: Hoof trimming by the farmer; HT: Hoof trimmer hired from outside.

**Table 2:** List of abbreviations for specific claw lesions and lesion types.

Abbreviations for Claw Lesions/Lesion Types	Description of Claw Lesions
C-deform	Claw deformations such as concave dorsal wall (= chronic laminitic claws), corkscrew claws, scissor claws, asymmetric claws
CHL-wexcor	Claw horn lesions without exposed sole or wall corium such as sole haemorrhage, double sole, white line lesion of severity score 1 (white line separation)
DD-ass	Dermatitis digitalis (DD)-associated claw horn lesions with exposed corium such as sole ulcer, toe ulcer, toe necrosis, bulb ulcer, white line abscess, perforating horn fissure (assumed as DD-ass as the cows were housed in farms with endemic DD-infection)
DD-M2	Acute (M2 stage) of digital dermatitis
DD-M1+M4	Early (M1) and chronic (M4) stages of digital dermatitis
HHE	Heel horn erosion (severity scores 2 and 3)
IH	Interdigital hyperplasia
IH-DD-M2	Interdigital hyperplasia with acute DD-M2 on their surface
Infect	Infectious claw disorders without DD such as inflammatory swelling of the coronet/bulb and interdigital phlegmon (foot rot)
O-caus	‘Other causes’ of lameness including a block being attached to a claw (in four cows on hindlimbs), or the presence of disorders in the proximal limb regions
TS	Thin sole

**Table 3:** List of cow prevalences of claw lesions and lesion types (with at least one of these lesions per cow) in 491 cows and overview of the distribution of severity scores 1–3 of the lesions. The abbreviations for the claw lesions are explained in the legend of Table 2.

Abbreviations for Claw Lesions/Lesion Types	Number of Cows (%)	Severity Score 1 %	Severity Score 2 %	Severity Score 3 %
C-deform	114 (23.2)	68.2	27.1	4.7
CHL-wexcor	285 (58.0)	73.7	21.7	4.6
DD-ass	51 (10.4)	No differentiation into different severity scores		
DD-M2 ^1^	75 (15.3)	No differentiation into different severity scores		
DD-M1+M4 ^2^	38 (7.7)	No differentiation into different severity scores		
HHE	267 (54.4)	−	98.9	1.1
IH	43 (8.7)	78.1	21.9	0.0
IH-DD-M2	27 (5.5)	0.0	37.0	63.0
Infect	9 (1.8)	22.2	55.6	22.2
O-caus	23 (4.7)	No differentiation into different severity scores		
TS	53 (10.8)	No differentiation into different severity scores		

^1^ Stage M2 of digital dermatitis (DD). ^2^ Stage M1 and M4 of DD, without concurrent occurrence of acute DD (M2) lesions in these cows.

**Table 4:** Overview of the number and location of claw lesions in all cows; LF LC = left forelimb lateral claw; LF MC = left forelimb medial claw; RF MC = right forelimb medial claw; RF LC = right forelimb lateral claw; LH LC = left hindlimb lateral claw; LH MC = left hindlimb medial claw; RH MC = right hindlimb medial claw; RH LC = right hindlimb lateral claw.

Localisation	LF LC	LF MC	RF MC	RF LC	LH LC	LH MC	RH MC	RH LC
Number	505	491	517	497	777	458	450	767

**Table 5:** List of the number and percentage (%) cows with documented claw lesions scored with LCS 1 (*n* = 200) and LCSs ≥ 2 (*n* = 291). The abbreviations for the claw lesions are explained in the legend of Table 2.

	C-deform (%)	CHL- wexcor (%)	DD-ass (%)	DD-M2 (%)	DD-M1+M4 (%)	HHE (%)	IH (%)	IH-DD-M2 (%)	Infect (%)	O-caus (%)	TS (%)
**LCS 1**	38 (19.0)	103 (51.5)	14 (7.0)	25 (12.5)	18 (9.0)	97 (48.5)	10 (5.0)	1 (0.5)	1 (0.5)	2 (1.0)	3 (1.5)
**LCS ≥ 2**	76 (26.1)	182 (62.5)	37 (12.7)	50 (17.2)	20 (6.9)	170 (58.4)	33 (11.3)	26 (8.9)	8 (2.7)	21 (7.2)	50 (17.2)

**Table 6:** List of the number and percentage (%) of ‘alarm’ lesions including DD-associated claw horn lesions (DD-ass), acute M2 digital dermatitis (DD-M2), interdigital hyperplasia with acute DD on the top (IH-DD-M2), infectious claw disorders (Infect: foot rot, inflammatory swelling of the coronet/bulb), and thin soles (TS) documented in the 491 cows and their distribution among the locomotion scores LCS 1, LCS 2, LCS 3 and LCS 4.

	Total	LCS 1	LCS 2	LCS 3	LCS 4
Number	185	43	78	42	22
(%)	(37.7) ^1^	(21.5) ^2^	(26.8) ^3^	(14.4) ^3^	(7.6) ^3^

^1^ The percentage was calculated based on the total number of all 491 cows. ^2^ The percentage was calculated based on the number of cows with LCS 1 (*n* = 200). ^3^ The percentage was calculated based on the number of cows with LCS ≥ 2 (*n* = 291).

**Table 7:** Results of the simple chi-square (χ^2^) test and the generalised linear mixed model (GLMM), corrected for the farm, lactation number, and lactation stage, between the respective claw lesions and locomotion score (LCS) ≥ 2, LCS ≥ 3, and LCS ≥ 4 [6] of the 491 cows; *: indicates a significant association; OR = odds ratio; 95% CI = 95% confidence interval. The abbreviations for the claw lesions are explained in the legend of Table 2.

		LCS ≥ 2	LCS ≥ 3	LCS ≥ 4
Claw Lesions		OR | 95% CI | *p*-Value	OR | 95% CI | *p*-Value	OR | 95% CI | *p*-Value
C-deform	χ^2^ test	1.5 | 1.0–2.3 | 0.0665	1.9 | 1.1–3.1 | 0.0138 *	2.2 | 0.9–5.3 | 0.0641
	GLMM	1.8 | 0.9–3.6 | 0.0720	1.5 | 0.6–3.6 | 0.3574	0.8 | 0.2–3.4 | 0.7689
CHL- wexcor	χ^2^ test	1.6 | 1.1–2.3 | 0.0148 *	1.9 | 1.1–3.1 | 0.0119 *	2.7 | 1.0–7.4 | 0.0442 *
	GLMM	1.0 | 0.6–1.7 | 0.8987	1.3 | 0.6–2.9 | 0.5037	1.9 | 0.4–8.4 | 0.4143
DD-ass	χ^2^ test	1.9 | 1.0–3.7 | 0.0414 *	4.4 | 2.4–8.1 | <0.0001 ^1^*	5.3 | 2.1–13.1 | <0.0001 ^1^*
	GLMM	1.5 | 0.7–3.3 | 0.2814	4.0 | 1.6–9.9 | 0.0032 *	5.3 | 1.3–21.2 | 0.0200 *
DD-M2	χ^2^ test	1.4 | 0.9–2.4 | 0.1565	1.6 | 0.9–2.9 | 0.1217	1.6 | 0.6–4.4 | 0.3774 ^1^
	GLMM	1.0 | 0.5–1.9 | 0.3785	1.3 | 0.5–3.1 | 0.6241	1.3 | 0.3–6.1 | 0.7010
DD1+DD4	χ^2^ test	0.7 | 0.4–1.4 | 0.3861	0.7 | 0.3–1.8 | 0.4433	na | na | 0.1548 ^1,2^
	GLMM	0.7 | 0.3–1.6 | 0.9617	1.1 | 0.3–3.7 | 0.9119	- ^3^
HHE	χ^2^ test	1.5 | 1.1–2.1 | 0.0301 *	2.1 | 1.3–3.5 | 0.0026 *	3.2 | 1.2–8.7 | 0.0185 *
	GLMM	0.9 | 0.5–1.6 | 0.7989	1.0 | 0.4–2.1 | 0.9055	1.7 | 0.3–8.3 | 0.5136
IH	χ^2^ test	2.4 | 1.2–5.1 | 0.0146 *	4.9 | 2.6–9.5 | <0.0001 *	3.1 | 1.1–8.9 | 0.0241 ^1^*
	GLMM	1.1 | 0.4–2.8 | 0.8373	3.0 | 1.2–7.5 | 0.0157 *	1.0 | 0.2–4.6 | 0.9936
IH-DD-M2	χ^2^ test	19.5 | 2.6–145.1 | <0.0001 *	6.8 | 3.1–15.1 | <0.0001 *	12.6 | 4.8–33.4 | <0.0001 ^1^*
	GLMM	17.6 | 2.1–148.5 | 0.0087 *	3.4 | 1.1–10.9 | 0.0367 *	9.1 | 2.3–36.4 | 0.0019 *
Infect	χ^2^ test	5.6 | 0.7–45.3 | 0.0679 ^1^	6.1 | 1.6–23.2 | 0.0027 ^1^*	19.5 | 4.8–78.5 | <0.0001 ^1^*
	GLMM	1.2 | 0.1–17.3 | 0.8683	1.8 | 0.3–10.8 | 0.4917	10.0 | 1.7–57.7 | 0.0102 *
O-caus	χ^2^ test	7.7 | 1.8–33.2 | 0.0014 *	3.9 | 1.7–9.2 | 0.0009 *	6.9 | 2.3–20.8 | <0.0001 ^1^*
	GLMM	6.3 | 1.2–32.0 | 0.0276 *	1.7 | 0.5–6.2 | 0.4044	2.8 | 0.6–12.8 | 0.1787
TS	χ^2^ test	13.6 | 4.2–44.3 | <0.0001 *	3.7 | 2.0–6.8 | <0.0001 *	2.4 | 0.9–6.8 | 0.0832 ^1^
	GLMM	19.8 | 2.0–197.2 | 0.0128 *	7.2 | 1.1–47.8 | 0.0424 *	3.2 | 0.1–128.5 | 0.4868

^1^ Requirements for χ^2^ test regarding cell counts not met, Fisher’s exact tests were additionally performed and resulted in similar *p*-values: for Infect and LCS ≥ 2 *p* = 0.0898; Infect and LCS ≥ 3 *p* = 0.0109; IH-DD-M2 and LCS ≥ 3 *p* < 0.0001; O-caus and LCS ≥ 4 *p* = 0.0028; DD-ass and LCS ≥ 4 *p* = 0.0011; Infect and LCS ≥ 4 *p* = 0.0004; DD-M2 and LCS ≥ 4 *p* = 0.3734; IH-DD-M2 and LCS ≥ 4 *p* < 0.0001; IH and LCS ≥ 4 *p* = 0.0416; TS and LCS ≥ 4 *p* = 0.0898; DD1+DD4 and LCS ≥ 4 *p* = 0.2426. ^2^ Not available (na) as no cases with DD1+DD4 occurred in combination with LCS ≥ 4. ^3^ As no cases with DD1+DD4 occurred in combination with LCS ≥ 4, the original model did not converge, and the effect had to be excluded.

**Table 8:** Crosstabulation of claw lesions in 491 cows from ten farms. The abbreviations for the claw lesions are explained in the legend of Table 2.

	C-deform (%)	CHL-wexcor (%)	DD-ass (%)	DD-M2 (%)	DD-M1 +M4 (%)	HHE (%)	IH (%)	IH-DD-M2 (%)	Infect (%)	O-caus (%)	TS (%)
C-deform (*n* = 114)		90 (79.0)	23 (20.2)	13 (11.4)	9 (7.9)	95 (83.3)	14 (12.3)	9 (7.9)	5 (4.4)	6 (5.3)	3 (2.6)
CHL-wexcor (*n* = 285)	90 (31.6)		33 (11.6)	34 (11.9)	19 (6.7)	178 (62.5)	35 (12.3)	20 (7.0)	7 (2.5)	15 (5.3)	38 (13.3)
DD-ass (*n* = 51)	23 (45.1)	33 (64.7)		3 (5.9)	2 (3.9)	36 (70.6)	5 (9.8)	1 (2.0)	3 (5.9)	7 (13.7)	5 (9.8)
DD-M2 (*n* = 75)	13 (17.3)	34 (45.3)	3 (4.0)		0 (0.0)	43 (57.3)	2 (2.7)	4 (5.3)	1 (1.3)	4 (5.3)	9 (12.0)
DD-M1+M4 (*n* = 38)	9 (23.7)	19 (50.0)	2 (5.3)	0 (0.0)		26 (68.4)	2 (5.3)	1 (2.6)	0 (0.0)	2 (5.3)	4 (10.5)
HHE (*n* = 267)	95 (35.6)	178 (66.7)	36 (13.5)	43 (16.1)	26 (9.7)		32 (12.0)	19 (7.1)	7 (2.6)	16 (6.0)	34 (12.7)
IH (*n* = 43)	14 (32.6)	35 (81.4)	5 (11.6)	2 (4.7)	2 (4.7)	32 (74.4)		6 (14.0)	1 (2.3)	7 (16.3)	8 (18.6)
IH-DD-M2 (*n* = 27)	9 (33.3)	20 (74.1)	1 (3.7)	4 (14.8)	1 (3.7)	19 (70.4)	6 (22.2)		1 (3.7)	3 (11.1)	2 (7.4)
Infect (*n* = 9)	5 (55.6)	7 (77.8)	3 (33.3)	1 (11.1)	0 (0.0)	7 (77.8)	1 (11.1)	1 (11.1)		2 (22.2)	2 (22.2)
O-caus (*n* = 23)	6 (26.1)	15 (65.2)	7 (30.4)	4 (17.4)	2 (8.7)	16 (69.6)	7 (30.4)	3 (13.0)	2 (8.7)		5 (21.7)
TS (*n* = 53)	3 (5.7)	38 (71.7)	5 (9.4)	9 (17.0)	4 (7.6)	34 (64.2)	8 (15.1)	2 (3.8)	2 (3.8)	5 (9.4)	

## Data Availability

The data used in the current study are not publicly available due to privacy restrictions of the patient data and the patient owners.
